# BMC Sports science, medicine, and rehabilitation reviewer acknowledgement 2014

**DOI:** 10.1186/2052-1847-7-6

**Published:** 2015-02-18

**Authors:** Clare Partridge

**Affiliations:** BioMed Central, Floor 6, 236 Gray’s Inn Road, London, WC1X 8HB UK

## Abstract

The editors of Sports Science, Medicine and Rehabilitation would like to thank all our reviewers who have contributed to the journal in Volume 6 (2014).

**Simon Angus**

Australia

**Francisco Areces Corcuera**

Spain

**Julien Steven Baker**

UK

**Arnhild Bakken**

Qatar

**Tiago Barbosa**

Singapore

**Alan Barker**

UK

**Alan Batterham**

UK

**Tom Bauer**

USA

**Britton Brewer**

USA

**Aurelien Bringard**

Switzerland

**Steven Broglio**

USA

**Martin Burtscher**

Austria

**Michael Carmont**

UK

**Adelaida María Castro-Sánchez**

Spain

**Karly Oi Wan Chan**

China

**Chi Ching Gary Chow**

Hong Kong

**James Clark**

USA

**Jamie Cooper**

USA

**Jennifer Copeland**

Canada

**Mario Costa**

Portugal

**Carlos Crestani**

Brazil

**Antonio Crisafulli**

Italy

**Aaron Daluiski**

USA

**Fred Dimenna**

USA

**Catherine Disselhorst-Klug**

Germany

**Lars Donath**

Switzerland

**Jacques Duysens**

Netherlands

**Pascal Edouard**

France

**Mikel Egana**

Ireland

**Brandon Erickson**

USA

**Jonathan Esteve-Lanao**

Spain

**Gertjan Ettema**

Norway

**Hani Fadel**

Saudi Arabia

**Abdulaziz Farooq**

Qatar

**Oliver Faude**

Switzerland

**Catherine Fieseler**

USA

**Richard Foster**

UK

**Reginaldo Fukuchi**

Brazil

**Alejandro Galan-Mercant**

Spain

**Laura Goldberg**

USA

**Eric Goulet**

Canada

**Mathieu Gourlan**

France

**Freedom Nkhululeko Gumedze**

South Africa

**Maria Hagstromer**

Sweden

**Brian Hanley**

UK

**Marquis Hawkins**

USA

**Stewart Head**

Australia

**Jose M Heredia Jimenez**

Spain

**Angela Hibbs**

UK

**John Hill**

USA

**Jonathan Hoffman**

Israel

**Kuno Hottenrott**

Germany

**Wen-Hao Hsu**

USA

**John Timothy Inglis**

Canada

**Athanasios Jamurtas**

Greece

**John Kalbfleisch**

USA

**Alon Kalron**

Israel

**Michelle Keightley**

Canada

**Michael Kingsley**

Australia

**Beat Knechtle**

Switzerland

**Kevin Kwong**

Hong Kong

**Joyce Cy Leung**

Hong Kong

**Carl Lombard**

South Africa

**Wings Tjing Yung Loo**

Hong Kong

**Reidar Lystad**

Australia

**Jelena Macan**

Croatia

**Duncan Macfarlane**

Hong Kong

**Fabiana Machado**

Brazil

**Zuzana Machotka**

Australia

**Nicola Maffulli**

UK

**Daniel Marinho**

Portugal

**Amy Mark**

Canada

**Fulvio Marzatico**

Italy

**Kei Masani**

Canada

**Michele Mauro**

Italy

**Alan McCall**

France

**Teri McCambridge**

USA

**Suzanne McDonough**

UK

**Chris McLellan**

Australia

**Jose Antonio Merchán-Baeza**

Spain

**Neil Messenger**

UK

**Guillaume Millet**

Canada

**Tony Monnet**

France

**Kevin Netto**

Australia

**Georg Neumann**

Germany

**Zhen Ni**

Canada

**Pantelis Nikolaidis**

Greece

**David Oxborough**

UK

**Adriana Perez**

USA

**Maria Francesca Piacentini**

Italy

**Pierre Portero**

France

**Geoff Power**

Canada

**Craig Ranson**

UK

**Christoph Raschka**

Germany

**Charles Rice**

Canada

**Gordon Robertson**

Canada

**Jose M Saavedra**

Spain

**Daniel Sánchez-Zuriaga**

Spain

**Jonathon Senefeld**

USA

**Mariano Serrao**

Italy

**Sameer Shah**

USA

**Jasper Shealy**

USA

**Tzyy-Yuang Shiang**

Taiwan

**Eric Stöhr**

UK

**Antoni Sureda**

Spain

**Wolfgang Taube**

Switzerland

**Pedro Tauler**

Spain

**Danny Taylor**

UK

**Daniel Theisen**

Luxembourg

**Panos Thomas**

UK

**Mike Tipton**

UK

**Takuro Tobina**

Japan

**Tamara Valovich Mcleod**

USA

**Vincent Van Hees**

UK

**Cahouet Violaine**

France

**Feng Wei**

USA

**Jeffrey Willardson**

USA

**Jonathan Williams**

UK

**Junjie Xiao**

China

**Ewa Ziemann**

Poland

**Hassane Zouhal**

France

